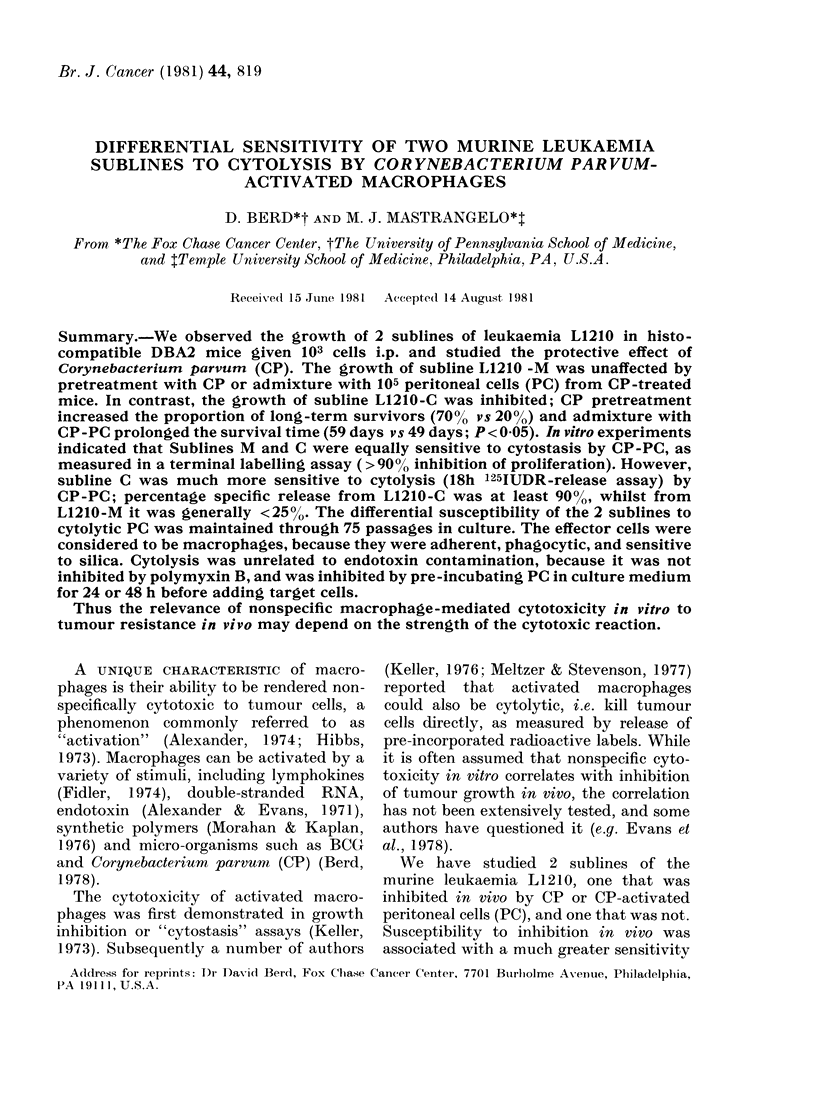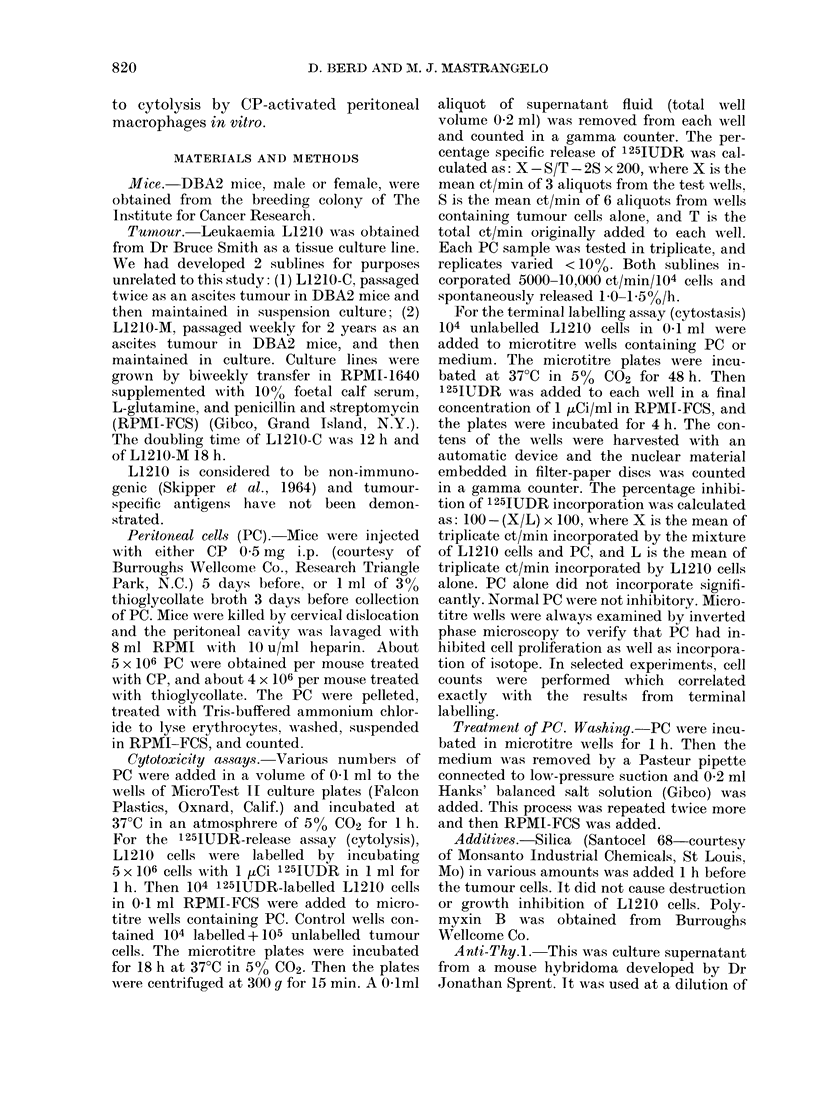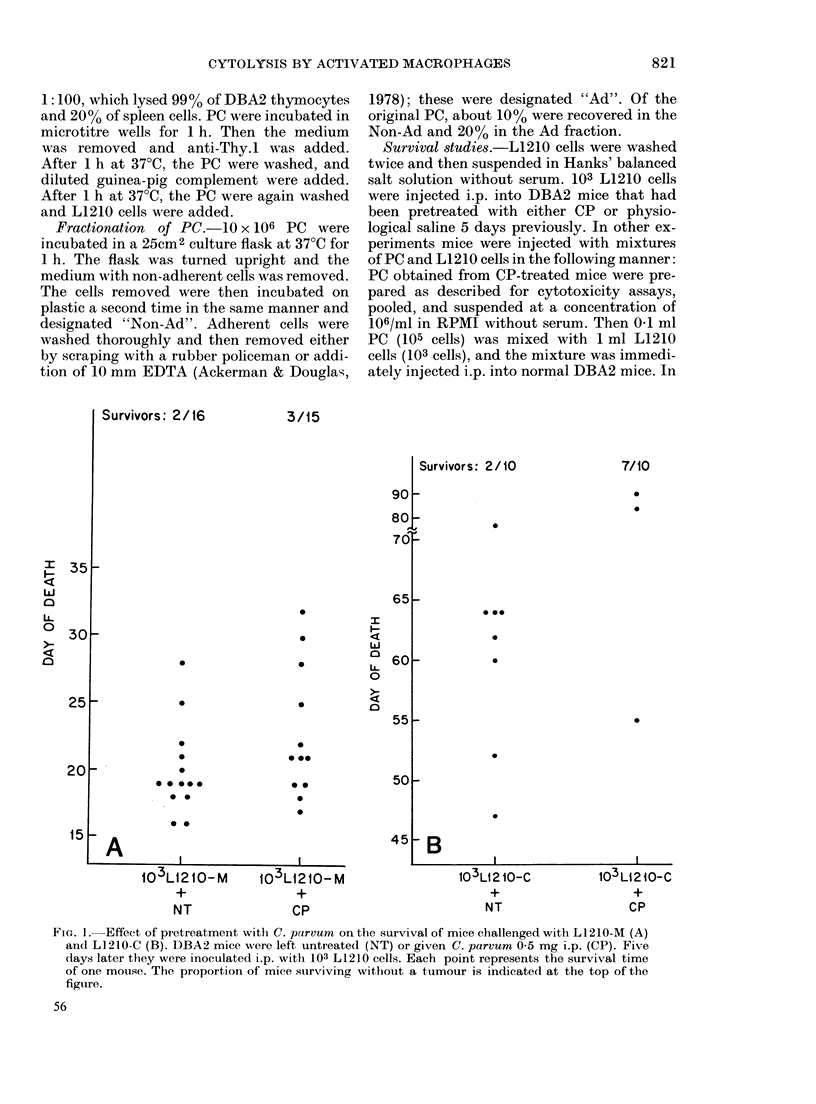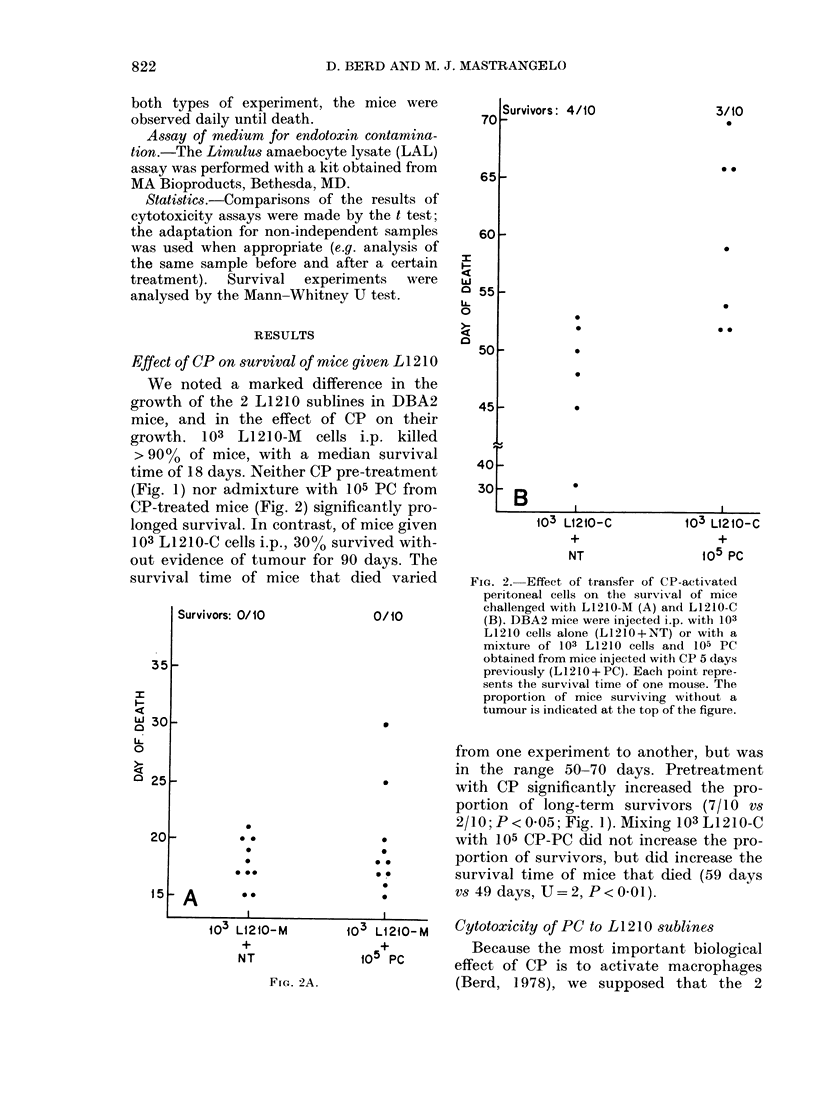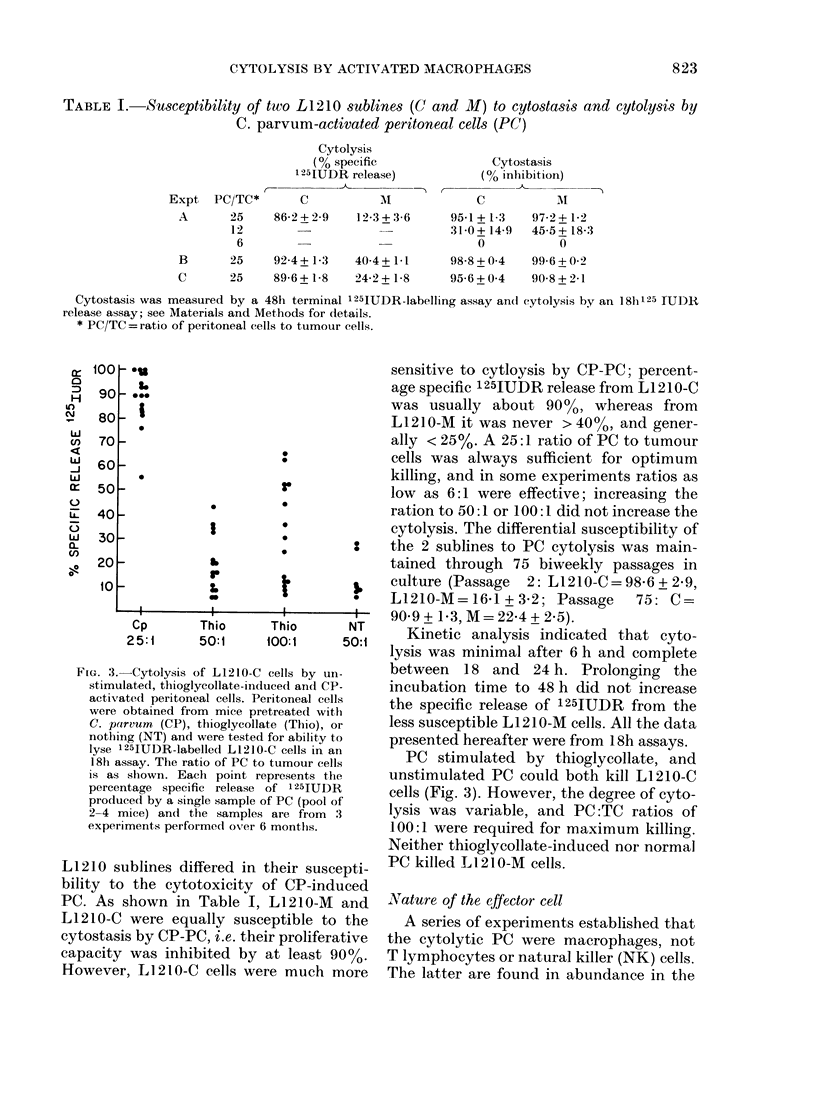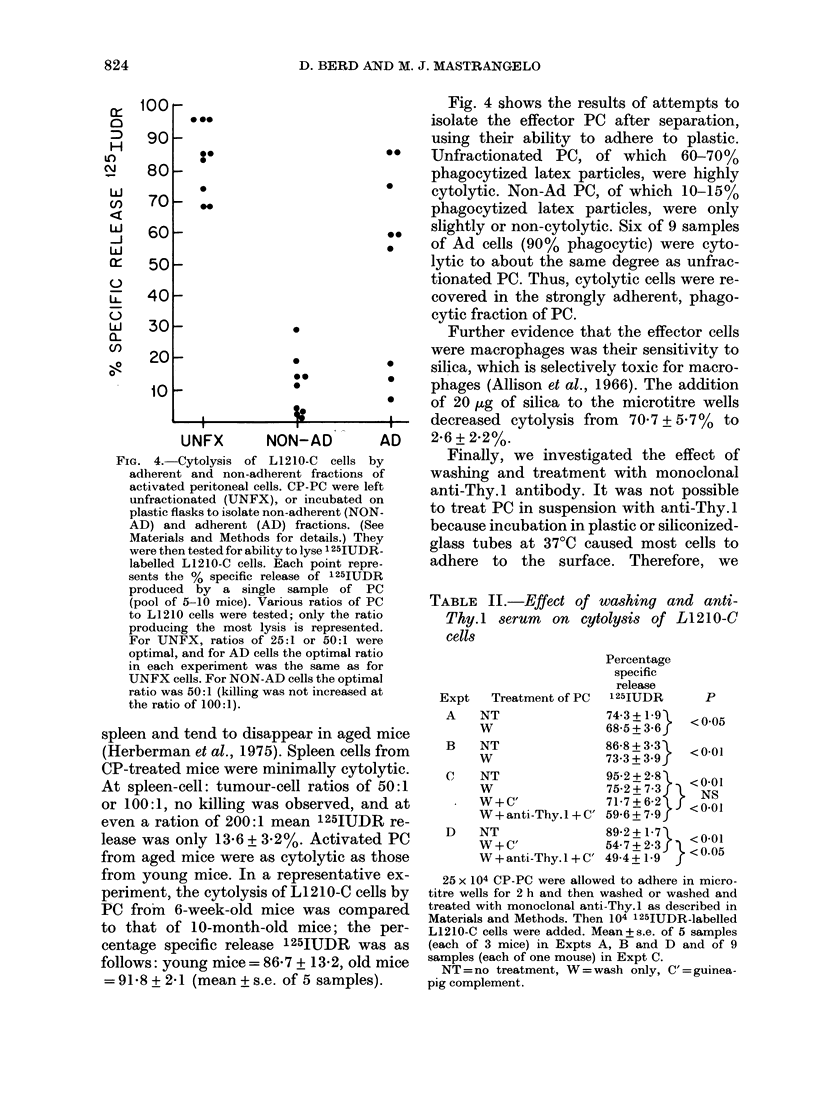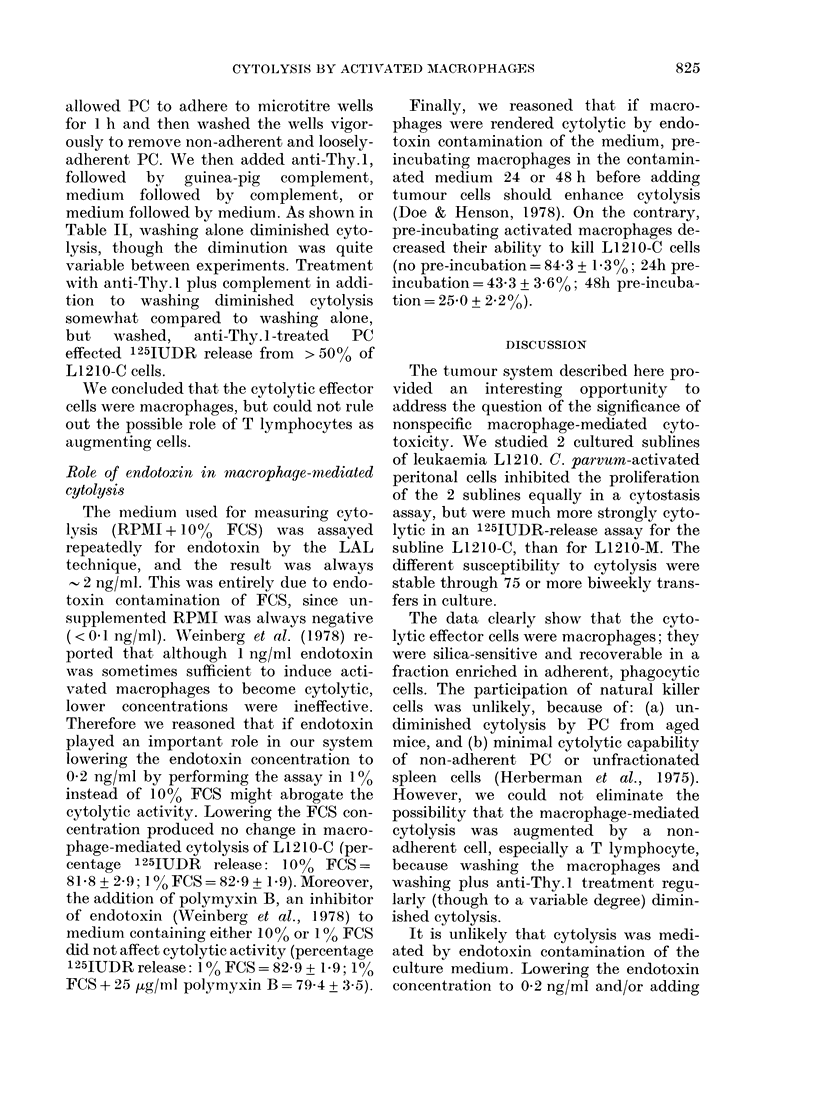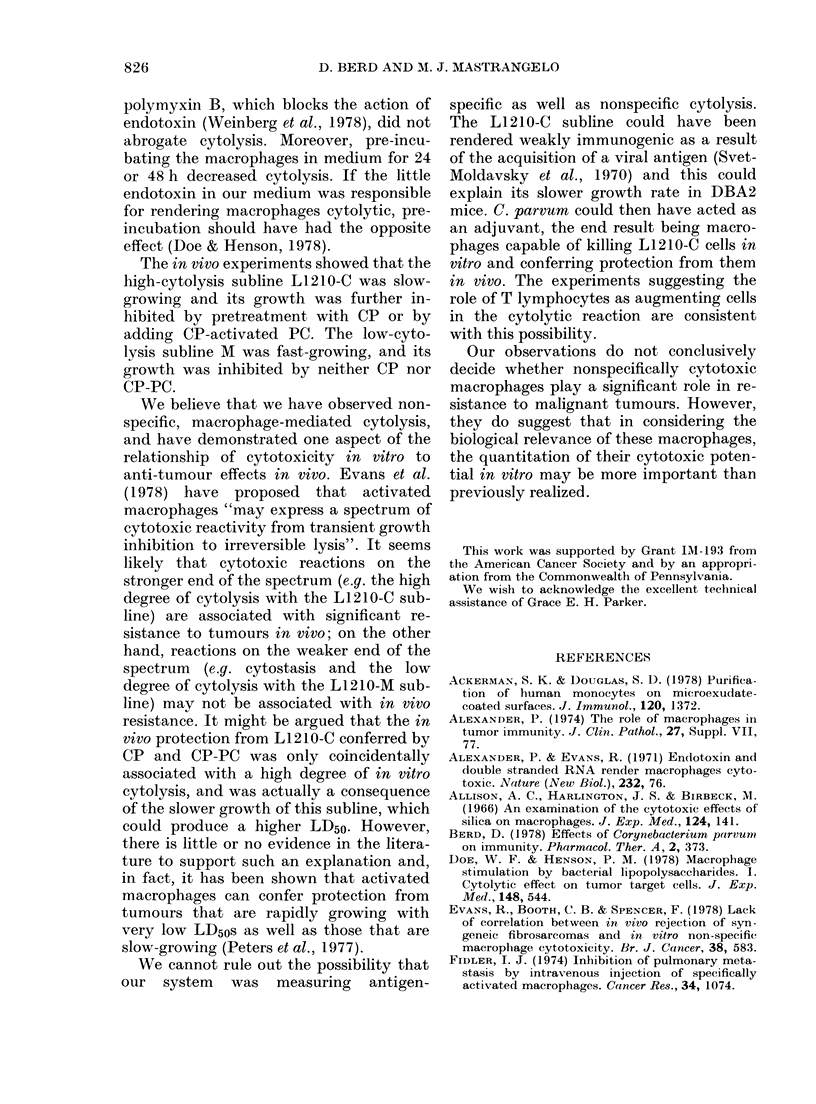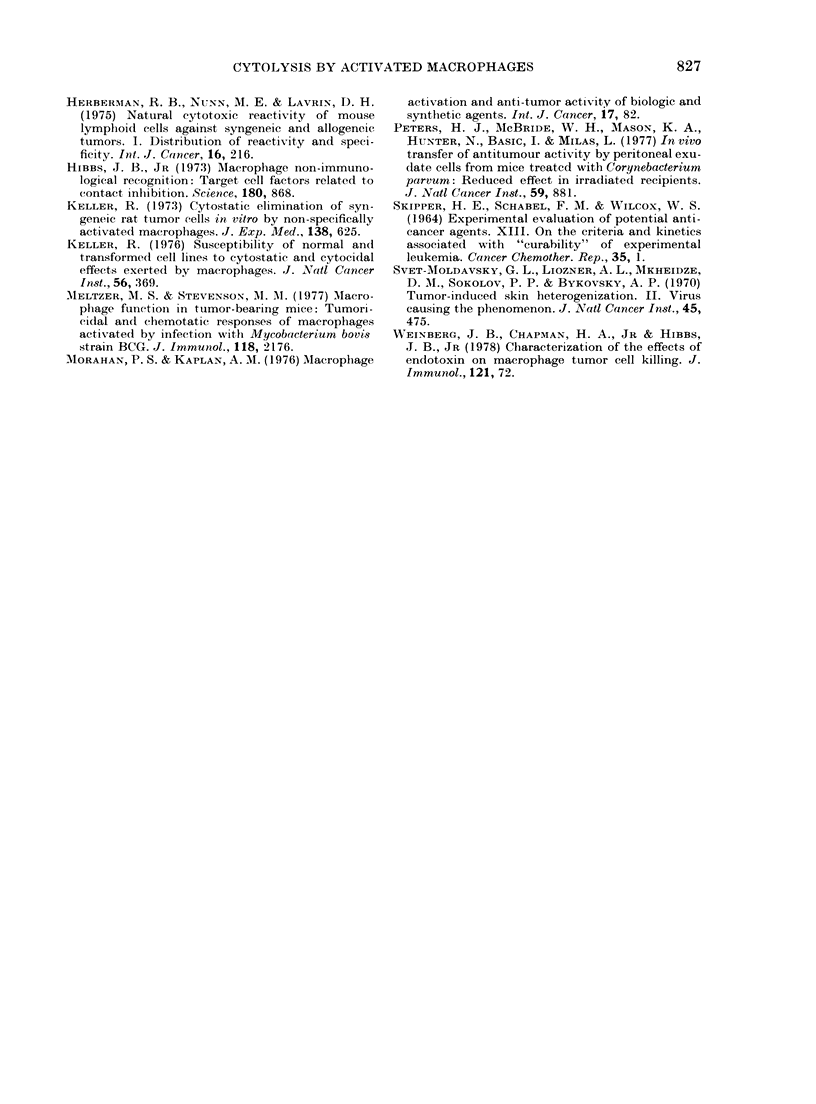# Differential sensitivity of two murine leukaemia sublines to cytolysis by Corynebacterium parvum-activated macrophages.

**DOI:** 10.1038/bjc.1981.280

**Published:** 1981-12

**Authors:** D. Berd, M. J. Mastrangelo

## Abstract

We observed the growth of 2 sublines of leukaemia L1210 in histocompatible DBA2 mice given 10(3) cells i.p. and studied the protective effect of Corynebacterium parvum (CP). The growth of subline L1210-M was unaffected by pretreatment with CP or admixture with 10(5) peritoneal cells (PC) from CP-treated mice. In contrast, the growth of subline L1210-C was inhibited; CP pretreatment increased the proportion of long-term survivors (70% vs 20%) and admixture with CP-PC prolonged the survival time (59 days vs 49 days; P less than 0.05). In vitro experiments indicated that Sublines M and C were equally sensitive to cytostasis by CP-PC, as measured in a terminal labelling assay (greater than 90% inhibition of proliferation). However, subline C was much more sensitive to cytolysis (18h 125IUDR-release assay) by CP-PC; percentage specific release from L1210-C was at least 90%, whilst from L1210-M it was generally less than 25%. The differential susceptibility of the 2 sublines to cytolytic PC was maintained through 75 passages in culture. The effector cells were considered to be macrophages, because they were adherent, phagocytic, and sensitive to silica. Cytolysis was unrelated to endotoxin contamination, because it was not inhibited by polymyxin B, and was inhibited by pre-incubating PC in culture medium for 24 or 48 h before adding target cells. Thus the relevance of nonspecific macrophage-mediated cytotoxicity in vitro to tumour resistance in vivo may depend on the strength of the cytotoxic reaction.


*Br. J. Cancer* (1981) **44**, 819

### DIFFERENTIAL SENSITIVITY OF TWO MURINE LEUKAEMIA SUBLINES TO CYTOLYSIS BY *CORYNEBACTERIUM PARVUM*-ACTIVATED MACROPHAGES

D. BERD*† AND M. J. MASTRANGELO*‡

*From *The Fox Chase Cancer Center, †The University of Pennsylvania School of Medicine, and ‡Temple University School of Medicine, Philadelphia, PA, U.S.A.*

Received 15 June 1981 Accepted 14 August 1981

**Summary.—We observed the growth of 2 sublines of leukaemia L1210 in histocompatible DBA2 mice given $10^3$ cells i.p. and studied the protective effect of *Corynebacterium parvum* (CP). The growth of subline L1210 -M was unaffected by pretreatment with CP or admixture with $10^5$ peritoneal cells (PC) from CP-treated mice. In contrast, the growth of subline L1210-C was inhibited; CP pretreatment increased the proportion of long-term survivors (70% *vs* 20%) and admixture with CP-PC prolonged the survival time (59 days *vs* 49 days; $P<0{\cdot}05$). *In vitro* experiments indicated that Sublines M and C were equally sensitive to cytostasis by CP-PC, as measured in a terminal labelling assay (>90% inhibition of proliferation). However, subline C was much more sensitive to cytolysis (18h $^{125}$IUDR-release assay) by CP-PC; percentage specific release from L1210-C was at least 90%, whilst from L1210-M it was generally <25%. The differential susceptibility of the 2 sublines to cytolytic PC was maintained through 75 passages in culture. The effector cells were considered to be macrophages, because they were adherent, phagocytic, and sensitive to silica. Cytolysis was unrelated to endotoxin contamination, because it was not inhibited by polymyxin B, and was inhibited by pre-incubating PC in culture medium for 24 or 48 h before adding target cells.**

**Thus the relevance of nonspecific macrophage-mediated cytotoxicity *in vitro* to tumour resistance *in vivo* may depend on the strength of the cytotoxic reaction.**

A UNIQUE CHARACTERISTIC of macrophages is their ability to be rendered nonspecifically cytotoxic to tumour cells, a phenomenon commonly referred to as "activation" (Alexander, 1974; Hibbs, 1973). Macrophages can be activated by a variety of stimuli, including lymphokines (Fidler, 1974), double-stranded RNA, endotoxin (Alexander & Evans, 1971), synthetic polymers (Morahan & Kaplan, 1976) and micro-organisms such as BCG and *Corynebacterium parvum* (CP) (Berd, 1978).

The cytotoxicity of activated macrophages was first demonstrated in growth inhibition or "cytostasis" assays (Keller, 1973). Subsequently a number of authors (Keller, 1976; Meltzer & Stevenson, 1977) reported that activated macrophages could also be cytolytic, *i.e.* kill tumour cells directly, as measured by release of pre-incorporated radioactive labels. While it is often assumed that nonspecific cytotoxicity *in vitro* correlates with inhibition of tumour growth *in vivo*, the correlation has not been extensively tested, and some authors have questioned it (*e.g.* Evans *et al.*, 1978).

We have studied 2 sublines of the murine leukaemia L1210, one that was inhibited *in vivo* by CP or CP-activated peritoneal cells (PC), and one that was not. Susceptibility to inhibition *in vivo* was associated with a much greater sensitivity

Address for reprints: Dr David Berd, Fox Chase Cancer Center, 7701 Burholme Avenue, Philadelphia, PA 19111, U.S.A.

to cytolysis by CP-activated peritoneal macrophages *in vitro*.

### MATERIALS AND METHODS

*Mice*.—DBA2 mice, male or female, were obtained from the breeding colony of The Institute for Cancer Research.

*Tumour*.—Leukaemia L1210 was obtained from Dr Bruce Smith as a tissue culture line. We had developed 2 sublines for purposes unrelated to this study: (1) L1210-C, passaged twice as an ascites tumour in DBA2 mice and then maintained in suspension culture; (2) L1210-M, passaged weekly for 2 years as an ascites tumour in DBA2 mice, and then maintained in culture. Culture lines were grown by biweekly transfer in RPMI-1640 supplemented with 10% foetal calf serum, L-glutamine, and penicillin and streptomycin (RPMI-FCS) (Gibco, Grand Island, N.Y.). The doubling time of L1210-C was 12 h and of L1210-M 18 h.

L1210 is considered to be non-immunogenic (Skipper *et al.*, 1964) and tumour-specific antigens have not been demonstrated.

*Peritoneal cells* (PC).—Mice were injected with either CP 0·5 mg i.p. (courtesy of Burroughs Wellcome Co., Research Triangle Park, N.C.) 5 days before, or 1 ml of 3% thioglycollate broth 3 days before collection of PC. Mice were killed by cervical dislocation and the peritoneal cavity was lavaged with 8 ml RPMI with 10 u/ml heparin. About $5 \times 10^6$ PC were obtained per mouse treated with CP, and about $4 \times 10^6$ per mouse treated with thioglycollate. The PC were pelleted, treated with Tris-buffered ammonium chloride to lyse erythrocytes, washed, suspended in RPMI–FCS, and counted.

*Cytotoxicity assays*.—Various numbers of PC were added in a volume of 0·1 ml to the wells of MicroTest II culture plates (Falcon Plastics, Oxnard, Calif.) and incubated at 37°C in an atmosphrere of 5% $CO_2$ for 1 h. For the $^{125}$IUDR-release assay (cytolysis), L1210 cells were labelled by incubating $5 \times 10^6$ cells with 1 $\mu$Ci $^{125}$IUDR in 1 ml for 1 h. Then $10^4$ $^{125}$IUDR-labelled L1210 cells in 0·1 ml RPMI-FCS were added to microtitre wells containing PC. Control wells contained $10^4$ labelled + $10^5$ unlabelled tumour cells. The microtitre plates were incubated for 18 h at 37°C in 5% $CO_2$. Then the plates were centrifuged at 300 *g* for 15 min. A 0·1ml aliquot of supernatant fluid (total well volume 0·2 ml) was removed from each well and counted in a gamma counter. The percentage specific release of $^{125}$IUDR was calculated as: $X - S/T - 2S \times 200$, where X is the mean ct/min of 3 aliquots from the test wells, S is the mean ct/min of 6 aliquots from wells containing tumour cells alone, and T is the total ct/min originally added to each well. Each PC sample was tested in triplicate, and replicates varied $< 10\%$. Both sublines incorporated 5000–10,000 ct/min/$10^4$ cells and spontaneously released 1·0–1·5%/h.

For the terminal labelling assay (cytostasis) $10^4$ unlabelled L1210 cells in 0·1 ml were added to microtitre wells containing PC or medium. The microtitre plates were incubated at 37°C in 5% $CO_2$ for 48 h. Then $^{125}$IUDR was added to each well in a final concentration of 1 $\mu$Ci/ml in RPMI-FCS, and the plates were incubated for 4 h. The contens of the wells were harvested with an automatic device and the nuclear material embedded in filter-paper discs was counted in a gamma counter. The percentage inhibition of $^{125}$IUDR incorporation was calculated as: $100 - (X/L) \times 100$, where X is the mean of triplicate ct/min incorporated by the mixture of L1210 cells and PC, and L is the mean of triplicate ct/min incorporated by L1210 cells alone. PC alone did not incorporate significantly. Normal PC were not inhibitory. Microtitre wells were always examined by inverted phase microscopy to verify that PC had inhibited cell proliferation as well as incorporation of isotope. In selected experiments, cell counts were performed which correlated exactly with the results from terminal labelling.

*Treatment of PC. Washing*.—PC were incubated in microtitre wells for 1 h. Then the medium was removed by a Pasteur pipette connected to low-pressure suction and 0·2 ml Hanks' balanced salt solution (Gibco) was added. This process was repeated twice more and then RPMI-FCS was added.

*Additives*.—Silica (Santocel 68—courtesy of Monsanto Industrial Chemicals, St Louis, Mo) in various amounts was added 1 h before the tumour cells. It did not cause destruction or growth inhibition of L1210 cells. Polymyxin B was obtained from Burroughs Wellcome Co.

*Anti-Thy*.1.—This was culture supernatant from a mouse hybridoma developed by Dr Jonathan Sprent. It was used at a dilution of

1:100, which lysed 99% of DBA2 thymocytes and 20% of spleen cells. PC were incubated in microtitre wells for 1 h. Then the medium was removed and anti-Thy.1 was added. After 1 h at 37°C, the PC were washed, and diluted guinea-pig complement were added. After 1 h at 37°C, the PC were again washed and L1210 cells were added.

*Fractionation of PC.*—$10 \times 10^6$ PC were incubated in a 25cm$^2$ culture flask at 37°C for 1 h. The flask was turned upright and the medium with non-adherent cells was removed. The cells removed were then incubated on plastic a second time in the same manner and designated "Non-Ad". Adherent cells were washed thoroughly and then removed either by scraping with a rubber policeman or addition of 10 mm EDTA (Ackerman & Douglas, 1978); these were designated "Ad". Of the original PC, about 10% were recovered in the Non-Ad and 20% in the Ad fraction.

*Survival studies.*—L1210 cells were washed twice and then suspended in Hanks' balanced salt solution without serum. $10^3$ L1210 cells were injected i.p. into DBA2 mice that had been pretreated with either CP or physiological saline 5 days previously. In other experiments mice were injected with mixtures of PC and L1210 cells in the following manner: PC obtained from CP-treated mice were prepared as described for cytotoxicity assays, pooled, and suspended at a concentration of $10^6$/ml in RPMI without serum. Then 0·1 ml PC ($10^5$ cells) was mixed with 1 ml L1210 cells ($10^3$ cells), and the mixture was immediately injected i.p. into normal DBA2 mice. In

FIG. 1.—Effect of pretreatment with *C. parvum* on the survival of mice challenged with L1210-M (A) and L1210-C (B). DBA2 mice were left untreated (NT) or given *C. parvum* 0·5 mg i.p. (CP). Five days later they were inoculated i.p. with $10^3$ L1210 cells. Each point represents the survival time of one mouse. The proportion of mice surviving without a tumour is indicated at the top of the figure.

both types of experiment, the mice were observed daily until death.

*Assay of medium for endotoxin contamination.*—The *Limulus* amaebocyte lysate (LAL) assay was performed with a kit obtained from MA Bioproducts, Bethesda, MD.

*Statistics.*—Comparisons of the results of cytotoxicity assays were made by the *t* test; the adaptation for non-independent samples was used when appropriate (*e.g.* analysis of the same sample before and after a certain treatment). Survival experiments were analysed by the Mann–Whitney U test.

### RESULTS

### *Effect of CP on survival of mice given L*1210

We noted a marked difference in the growth of the 2 L1210 sublines in DBA2 mice, and in the effect of CP on their growth. $10^3$ L1210-M cells i.p. killed $>90\%$ of mice, with a median survival time of 18 days. Neither CP pre-treatment (Fig. 1) nor admixture with $10^5$ PC from CP-treated mice (Fig. 2) significantly prolonged survival. In contrast, of mice given $10^3$ L1210-C cells i.p., 30% survived without evidence of tumour for 90 days. The survival time of mice that died varied from one experiment to another, but was in the range 50–70 days. Pretreatment with CP significantly increased the proportion of long-term survivors (7/10 *vs* 2/10; $P<0{\cdot}05$; Fig. 1). Mixing $10^3$ L1210-C with $10^5$ CP-PC did not increase the proportion of survivors, but did increase the survival time of mice that died (59 days *vs* 49 days, $U=2$, $P<0{\cdot}01$).

FIG. 2A.

FIG. 2.—Effect of transfer of CP-activated peritoneal cells on the survival of mice challenged with L1210-M (A) and L1210-C (B). DBA2 mice were injected i.p. with $10^3$ L1210 cells alone (L1210+NT) or with a mixture of $10^3$ L1210 cells and $10^5$ PC obtained from mice injected with CP 5 days previously (L1210+PC). Each point represents the survival time of one mouse. The proportion of mice surviving without a tumour is indicated at the top of the figure.

### *Cytotoxicity of PC to L*1210 *sublines*

Because the most important biological effect of CP is to activate macrophages (Berd, 1978), we supposed that the 2

TABLE I.—*Susceptibility of two L1210 sublines (C and M) to cytostasis and cytolysis by* C. parvum-*activated peritoneal cells (PC)*

| | | Cytolysis (% specific $^{125}$IUDR release) | | Cytostasis (% inhibition) | |
|---|---|---|---|---|---|
| Expt | PC/TC* | C | M | C | M |
| A | 25 | 86·2 ± 2·9 | 12·3 ± 3·6 | 95·1 ± 1·3 | 97·2 ± 1·2 |
| | 12 | — | — | 31·0 ± 14·9 | 45·5 ± 18·3 |
| | 6 | — | — | 0 | 0 |
| B | 25 | 92·4 ± 1·3 | 40·4 ± 1·1 | 98·8 ± 0·4 | 99·6 ± 0·2 |
| C | 25 | 89·6 ± 1·8 | 24·2 ± 1·8 | 95·6 ± 0·4 | 90·8 ± 2·1 |

Cytostasis was measured by a 48h terminal $^{125}$IUDR-labelling assay and cytolysis by an 18h $^{125}$IUDR release assay; see Materials and Methods for details.
* PC/TC = ratio of peritoneal cells to tumour cells.

FIG. 3.—Cytolysis of L1210-C cells by unstimulated, thioglycollate-induced and CP-activated peritoneal cells. Peritoneal cells were obtained from mice pretreated with *C. parvum* (CP), thioglycollate (Thio), or nothing (NT) and were tested for ability to lyse $^{125}$IUDR-labelled L1210-C cells in an 18h assay. The ratio of PC to tumour cells is as shown. Each point represents the percentage specific release of $^{125}$IUDR produced by a single sample of PC (pool of 2–4 mice) and the samples are from 3 experiments performed over 6 months.

L1210 sublines differed in their susceptibility to the cytotoxicity of CP-induced PC. As shown in Table I, L1210-M and L1210-C were equally susceptible to the cytostasis by CP-PC, *i.e.* their proliferative capacity was inhibited by at least 90%. However, L1210-C cells were much more sensitive to cytloysis by CP-PC; percentage specific $^{125}$IUDR release from L1210-C was usually about 90%, whereas from L1210-M it was never > 40%, and generally < 25%. A 25:1 ratio of PC to tumour cells was always sufficient for optimum killing, and in some experiments ratios as low as 6:1 were effective; increasing the ration to 50:1 or 100:1 did not increase the cytolysis. The differential susceptibility of the 2 sublines to PC cytolysis was maintained through 75 biweekly passages in culture (Passage 2: L1210-C = 98·6 ± 2·9, L1210-M = 16·1 ± 3·2; Passage 75: C = 90·9 ± 1·3, M = 22·4 ± 2·5).

Kinetic analysis indicated that cytolysis was minimal after 6 h and complete between 18 and 24 h. Prolonging the incubation time to 48 h did not increase the specific release of $^{125}$IUDR from the less susceptible L1210-M cells. All the data presented hereafter were from 18h assays.

PC stimulated by thioglycollate, and unstimulated PC could both kill L1210-C cells (Fig. 3). However, the degree of cytolysis was variable, and PC:TC ratios of 100:1 were required for maximum killing. Neither thioglycollate-induced nor normal PC killed L1210-M cells.

### *Nature of the effector cell*

A series of experiments established that the cytolytic PC were macrophages, not T lymphocytes or natural killer (NK) cells. The latter are found in abundance in the

FIG. 4.—Cytolysis of L1210-C cells by adherent and non-adherent fractions of activated peritoneal cells. CP-PC were left unfractionated (UNFX), or incubated on plastic flasks to isolate non-adherent (NON-AD) and adherent (AD) fractions. (See Materials and Methods for details.) They were then tested for ability to lyse $^{125}$IUDR-labelled L1210-C cells. Each point represents the % specific release of $^{125}$IUDR produced by a single sample of PC (pool of 5–10 mice). Various ratios of PC to L1210 cells were tested; only the ratio producing the most lysis is represented. For UNFX, ratios of 25:1 or 50:1 were optimal, and for AD cells the optimal ratio in each experiment was the same as for UNFX cells. For NON-AD cells the optimal ratio was 50:1 (killing was not increased at the ratio of 100:1).

spleen and tend to disappear in aged mice (Herberman *et al.*, 1975). Spleen cells from CP-treated mice were minimally cytolytic. At spleen-cell: tumour-cell ratios of 50:1 or 100:1, no killing was observed, and at even a ration of 200:1 mean $^{125}$IUDR release was only 13·6 ± 3·2%. Activated PC from aged mice were as cytolytic as those from young mice. In a representative experiment, the cytolysis of L1210-C cells by PC from 6-week-old mice was compared to that of 10-month-old mice; the percentage specific release $^{125}$IUDR was as follows: young mice = 86·7 ± 13·2, old mice = 91·8 ± 2·1 (mean ± s.e. of 5 samples).

Fig. 4 shows the results of attempts to isolate the effector PC after separation, using their ability to adhere to plastic. Unfractionated PC, of which 60–70% phagocytized latex particles, were highly cytolytic. Non-Ad PC, of which 10–15% phagocytized latex particles, were only slightly or non-cytolytic. Six of 9 samples of Ad cells (90% phagocytic) were cytolytic to about the same degree as unfractionated PC. Thus, cytolytic cells were recovered in the strongly adherent, phagocytic fraction of PC.

Further evidence that the effector cells were macrophages was their sensitivity to silica, which is selectively toxic for macrophages (Allison *et al.*, 1966). The addition of 20 $\mu$g of silica to the microtitre wells decreased cytolysis from 70·7 ± 5·7% to 2·6 ± 2·2%.

Finally, we investigated the effect of washing and treatment with monoclonal anti-Thy.1 antibody. It was not possible to treat PC in suspension with anti-Thy.1 because incubation in plastic or siliconized-glass tubes at 37°C caused most cells to adhere to the surface. Therefore, we

TABLE II.—*Effect of washing and anti-Thy.*1 *serum on cytolysis of L*1210-*C cells*

| Expt | Treatment of PC | Percentage specific release $^{125}$IUDR | *P* |
|---|---|---|---|
| A | NT | 74·3 ± 1·9 | < 0·05 |
| | W | 68·5 ± 3·6 | |
| B | NT | 86·8 ± 3·3 | < 0·01 |
| | W | 73·3 ± 3·9 | |
| C | NT | 95·2 ± 2·8 | < 0·01 |
| | W | 75·2 ± 7·3 | NS |
| | W + C′ | 71·7 ± 6·2 | < 0·01 |
| | W + anti-Thy.1 + C′ | 59·6 ± 7·9 | |
| D | NT | 89·2 ± 1·7 | < 0·01 |
| | W + C′ | 54·7 ± 2·3 | < 0.05 |
| | W + anti-Thy.1 + C′ | 49·4 ± 1·9 | |

25 × 10$^4$ CP-PC were allowed to adhere in microtitre wells for 2 h and then washed or washed and treated with monoclonal anti-Thy.1 as described in Materials and Methods. Then 10$^4$ $^{125}$IUDR-labelled L1210-C cells were added. Mean ± s.e. of 5 samples (each of 3 mice) in Expts A, B and D and of 9 samples (each of one mouse) in Expt C.

NT = no treatment, W = wash only, C′ = guinea-pig complement.

allowed PC to adhere to microtitre wells for 1 h and then washed the wells vigorously to remove non-adherent and loosely-adherent PC. We then added anti-Thy.1, followed by guinea-pig complement, medium followed by complement, or medium followed by medium. As shown in Table II, washing alone diminished cytolysis, though the diminution was quite variable between experiments. Treatment with anti-Thy.1 plus complement in addition to washing diminished cytolysis somewhat compared to washing alone, but washed, anti-Thy.1-treated PC effected $^{125}$IUDR release from $>50\%$ of L1210-C cells.

We concluded that the cytolytic effector cells were macrophages, but could not rule out the possible role of T lymphocytes as augmenting cells.

*Role of endotoxin in macrophage-mediated cytolysis*

The medium used for measuring cytolysis (RPMI + 10% FCS) was assayed repeatedly for endotoxin by the LAL technique, and the result was always $\sim 2$ ng/ml. This was entirely due to endotoxin contamination of FCS, since unsupplemented RPMI was always negative ($<0{\cdot}1$ ng/ml). Weinberg *et al.* (1978) reported that although 1 ng/ml endotoxin was sometimes sufficient to induce activated macrophages to become cytolytic, lower concentrations were ineffective. Therefore we reasoned that if endotoxin played an important role in our system lowering the endotoxin concentration to 0·2 ng/ml by performing the assay in 1% instead of 10% FCS might abrogate the cytolytic activity. Lowering the FCS concentration produced no change in macrophage-mediated cytolysis of L1210-C (percentage $^{125}$IUDR release: 10% FCS = $81{\cdot}8 \pm 2{\cdot}9$; 1% FCS = $82{\cdot}9 \pm 1{\cdot}9$). Moreover, the addition of polymyxin B, an inhibitor of endotoxin (Weinberg *et al.*, 1978) to medium containing either 10% or 1% FCS did not affect cytolytic activity (percentage $^{125}$IUDR release: 1% FCS = $82{\cdot}9 \pm 1{\cdot}9$; 1% FCS + 25 $\mu$g/ml polymyxin B = $79{\cdot}4 \pm 3{\cdot}5$).

Finally, we reasoned that if macrophages were rendered cytolytic by endotoxin contamination of the medium, pre-incubating macrophages in the contaminated medium 24 or 48 h before adding tumour cells should enhance cytolysis (Doe & Henson, 1978). On the contrary, pre-incubating activated macrophages decreased their ability to kill L1210-C cells (no pre-incubation = $84{\cdot}3 \pm 1{\cdot}3\%$; 24h pre-incubation = $43{\cdot}3 \pm 3{\cdot}6\%$; 48h pre-incubation = $25{\cdot}0 \pm 2{\cdot}2\%$).

### DISCUSSION

The tumour system described here provided an interesting opportunity to address the question of the significance of nonspecific macrophage-mediated cytotoxicity. We studied 2 cultured sublines of leukaemia L1210. *C. parvum*-activated peritonal cells inhibited the proliferation of the 2 sublines equally in a cytostasis assay, but were much more strongly cytolytic in an $^{125}$IUDR-release assay for the subline L1210-C, than for L1210-M. The different susceptibility to cytolysis were stable through 75 or more biweekly transfers in culture.

The data clearly show that the cytolytic effector cells were macrophages; they were silica-sensitive and recoverable in a fraction enriched in adherent, phagocytic cells. The participation of natural killer cells was unlikely, because of: (a) undiminished cytolysis by PC from aged mice, and (b) minimal cytolytic capability of non-adherent PC or unfractionated spleen cells (Herberman *et al.*, 1975). However, we could not eliminate the possibility that the macrophage-mediated cytolysis was augmented by a non-adherent cell, especially a T lymphocyte, because washing the macrophages and washing plus anti-Thy.1 treatment regularly (though to a variable degree) diminished cytolysis.

It is unlikely that cytolysis was mediated by endotoxin contamination of the culture medium. Lowering the endotoxin concentration to 0·2 ng/ml and/or adding polymyxin B, which blocks the action of endotoxin (Weinberg *et al.*, 1978), did not abrogate cytolysis. Moreover, pre-incubating the macrophages in medium for 24 or 48 h decreased cytolysis. If the little endotoxin in our medium was responsible for rendering macrophages cytolytic, pre-incubation should have had the opposite effect (Doe & Henson, 1978).

The *in vivo* experiments showed that the high-cytolysis subline L1210-C was slow-growing and its growth was further inhibited by pretreatment with CP or by adding CP-activated PC. The low-cytolysis subline M was fast-growing, and its growth was inhibited by neither CP nor CP-PC.

We believe that we have observed non-specific, macrophage-mediated cytolysis, and have demonstrated one aspect of the relationship of cytotoxicity *in vitro* to anti-tumour effects *in vivo*. Evans *et al.* (1978) have proposed that activated macrophages "may express a spectrum of cytotoxic reactivity from transient growth inhibition to irreversible lysis". It seems likely that cytotoxic reactions on the stronger end of the spectrum (*e.g.* the high degree of cytolysis with the L1210-C subline) are associated with significant resistance to tumours *in vivo*; on the other hand, reactions on the weaker end of the spectrum (*e.g.* cytostasis and the low degree of cytolysis with the L1210-M subline) may not be associated with *in vivo* resistance. It might be argued that the *in vivo* protection from L1210-C conferred by CP and CP-PC was only coincidentally associated with a high degree of *in vitro* cytolysis, and was actually a consequence of the slower growth of this subline, which could produce a higher $LD_{50}$. However, there is little or no evidence in the literature to support such an explanation and, in fact, it has been shown that activated macrophages can confer protection from tumours that are rapidly growing with very low $LD_{50}$s as well as those that are slow-growing (Peters *et al.*, 1977).

We cannot rule out the possibility that our system was measuring antigen-specific as well as nonspecific cytolysis. The L1210-C subline could have been rendered weakly immunogenic as a result of the acquisition of a viral antigen (Svet-Moldavsky *et al.*, 1970) and this could explain its slower growth rate in DBA2 mice. *C. parvum* could then have acted as an adjuvant, the end result being macrophages capable of killing L1210-C cells *in vitro* and conferring protection from them *in vivo*. The experiments suggesting the role of T lymphocytes as augmenting cells in the cytolytic reaction are consistent with this possibility.

Our observations do not conclusively decide whether nonspecifically cytotoxic macrophages play a significant role in resistance to malignant tumours. However, they do suggest that in considering the biological relevance of these macrophages, the quantitation of their cytotoxic potential *in vitro* may be more important than previously realized.

This work was supported by Grant IM-193 from the American Cancer Society and by an appropriation from the Commonwealth of Pennsylvania.

We wish to acknowledge the excellent technical assistance of Grace E. H. Parker.

### REFERENCES

Ackerman, S. K. & Douglas, S. D. (1978) Purification of human monocytes on microexudate-coated surfaces. *J. Immunol.*, **120**, 1372.

Alexander, P. (1974) The role of macrophages in tumor immunity. *J. Clin. Pathol.*, **27**, Suppl. VII, 77.

Alexander, P. & Evans, R. (1971) Endotoxin and double stranded RNA render macrophages cytotoxic. *Nature* (*New Biol.*), **232**, 76.

Allison, A. C., Harlington, J. S. & Birbeck, M. (1966) An examination of the cytotoxic effects of silica on macrophages. *J. Exp. Med.*, **124**, 141.

Berd, D. (1978) Effects of *Corynebacterium parvum* on immunity. *Pharmacol. Ther. A*, **2**, 373.

Doe, W. F. & Henson, P. M. (1978) Macrophage stimulation by bacterial lipopolysaccharides. I. Cytolytic effect on tumor target cells. *J. Exp. Med.*, **148**, 544.

Evans, R., Booth, C. B. & Spencer, F. (1978) Lack of correlation between *in vivo* rejection of syngeneic fibrosarcomas and *in vitro* non-specific macrophage cytotoxicity. *Br. J. Cancer*, **38**, 583.

Fidler, I. J. (1974) Inhibition of pulmonary metastasis by intravenous injection of specifically activated macrophages. *Cancer Res.*, **34**, 1074.

HERBERMAN, R. B., NUNN, M. E. & LAVRIN, D. H. (1975) Natural cytotoxic reactivity of mouse lymphoid cells against syngeneic and allogeneic tumors. I. Distribution of reactivity and specificity. *Int. J. Cancer*, **16,** 216.

HIBBS, J. B., JR (1973) Macrophage non-immunological recognition: Target cell factors related to contact inhibition. *Science*, **180,** 868.

KELLER, R. (1973) Cytostatic elimination of syngeneic rat tumor cells *in vitro* by non-specifically activated macrophages. *J. Exp. Med.*, **138,** 625.

KELLER, R. (1976) Susceptibility of normal and transformed cell lines to cytostatic and cytocidal effects exerted by macrophages. *J. Natl Cancer Inst.*, **56,** 369.

MELTZER, M. S. & STEVENSON, M. M. (1977) Macrophage function in tumor-bearing mice: Tumoricidal and chemotatic responses of macrophages activated by infection with *Mycobacterium bovis* strain BCG. *J. Immunol.*, **118,** 2176.

MORAHAN, P. S. & KAPLAN, A. M. (1976) Macrophage activation and anti-tumor activity of biologic and synthetic agents. *Int. J. Cancer*, **17,** 82.

PETERS, H. J., MCBRIDE, W. H., MASON, K. A., HUNTER, N., BASIC, I. & MILAS, L. (1977) *In vivo* transfer of antitumour activity by peritoneal exudate cells from mice treated with *Corynebacterium parvum*: Reduced effect in irradiated recipients. *J. Natl Cancer Inst.*, **59,** 881.

SKIPPER, H. E., SCHABEL, F. M. & WILCOX, W. S. (1964) Experimental evaluation of potential anticancer agents. XIII. On the criteria and kinetics associated with "curability" of experimental leukemia. *Cancer Chemother. Rep.*, **35,** 1.

SVET-MOLDAVSKY, G. L., LIOZNER, A. L., MKHEIDZE, D. M., SOKOLOV, P. P. & BYKOVSKY, A. P. (1970) Tumor-induced skin heterogenization. II. Virus causing the phenomenon. *J. Natl Cancer Inst.*, **45,** 475.

WEINBERG, J. B., CHAPMAN, H. A., JR & HIBBS, J. B., JR (1978) Characterization of the effects of endotoxin on macrophage tumor cell killing. *J. Immunol.*, **121,** 72.